# Neurologic Diagnosis—Some First Principles

**Published:** 1912-01

**Authors:** Hansell Crenshaw

**Affiliations:** Atlanta, Ga.; Neurologist to the Wesley Memorial Hospital


### NEUROLOGIC DIAGNOSIS—SOME FIRST PRINCIPLES.

By Hansell Crenshaw, M. D., Atlanta, Ga.

*Neurologist to the Wesley Memorial Hospital.*

Obviously, there is neither need nor space for a systematic survey of the diagnosis of nervous diseases in a brief paper such as is here presented. Accordingly, I shall limit myself to a cogent grouping of a few first principles which I believe physicians generally need to get clearly and firmly fixed in mind.

First and fundamental in the diagnois of any nervous disease should be the determination of whether the disorder is organic, functional, or both. Only by this comprehensive preliminary step may we protect ourselves against the pitfalls of snap-judgment and occasional gross errors of diagnosis, prognosis and treatment. However unsatisfactory from the viewpoint of pathology may seem the division of neuroses into organic, or those showing structural changes, and "functional," or those unaccompanied by visible anatomical changes, the division is of prime importance to the diagnostician. Whether or not the so-called functional neuroses are dependent upon faulty nutrition of the neurons, only time and further study may determine.

It is perhaps best to assume every case to be organic until a searching investigation negatives this assumption. Not only may hysteria and neurasthenia simulate organic disease closely, but oftentimes they co-exist with organic diseases. The unwary diagnostician observing the obvious stigmata and accidents of hysteria, for example, in a mixed case is very likely to overlook the existence of the accompanying organic disease altogether.

Let me now briefly summarize the signs of organic nervous disease:

(1.) Localized convulsions are always caused by a corresponding irritation in the cerebral cortex—an irritation almost invariably due to organic disease.

(2.) Either spastic or tonic stiffening of the lower limbs is of organic origin if, when the examiner lifts one of the patient's feet, the pelvis and other leg are raised from the bed. This condition is called spinal epilepsy.

(3.) A persistent ankle-clonus, the vibrations of which are more than five per second and of uniform rate is highly indicative of organic disease. But a slow, irregular, transitory clonus may be hysterical.

(4.) Actual absence of the knee-jerk is almost proof positive of organic disease of either the nervous system or the muscles involved. Very rarely, however, it is met with in health and, we are told, it has been successfully simulated.

(5.) Atrophy of muscles accompanied by absence of Faradic irritability is either the result of organic nervous disease or of idiopathic disease of the muscles.

(6.) Paralysis affecting only the muscles supplied by the nerves from a given spinal segment is of organic origin, regardless of whether there is corresponding sensory loss or not. And both the so-called mowing gait and steppage gait are indicative of degeneration.

(7.) As is well known, permanent paralysis of the spinchters is always a certain sign of organic disease, and absence of control of the bladder and rectum is generally organic in origin.

(8.) Unilateral paralysis of the laryngeal muscles is certainly organic, though bilateral laryngeal paralysis may be functional.

(9.) Paralysis of the palate is generally an indication of organic disease, particularly oncoming diphtheritic paralysis.

(10.) Unless caused by uremia, paralysis of the face may be said to be always the result of organic disease.

(11.) Hemianopia, if clearly defined and permanent, is always organic, while either optic atrophy or optic neuritis is obviously an organic nervous disease. Although inequality in the size of the pupils should lead us to suspect organic brain lesions, this condition may occur in traumatic neurasthenia and other functional neuroses. The Argyll-Robertson pupil, however, is absolute proof of degeneration of the brain. Paralysis of the extrinsic muscle of the eyeball is due to organic brain disease when not the result of local conditions in the orbit, except those cases in which the paralysis is limited to the voluntary conjugate movements of both eyes.

It has been pointed out by Pershing that the absence of positive signs of organic disease does not always warrant us in assuming that we are dealing only with functional conditions. "If a delicate child has suffered for a week from headache with occasional vomiting," says Pershing, "the absence of all signs characteristic of organic disease requires the diagnosis of meningitis to be withheld but does not exclude it, for within the next few days pupillary changes, strabismus or some other conclusive symptom may appear." If, however, the positive signs of organic disease do not appear after a considerable period, say two or three months, the exclusion of an organic basis for the trouble may reasonably be made.

After having ascertained whether a given case is organic or functional, our next business is, of course, to set about a more special classification of it. Let us consider first what our course of reasoning should be in respect of an organic case. Gowers divides organic nervous diseases into vascular lesions, inflammations, degenerations and growths. We may generally gain an immediate insight into the nature of our case by a study of the rate of onset. Roughly speaking, cases with an onset varying from a few minutes to a few hours are vascular—that is, either hemorrhages, embolisms, or thromboses. Cases developing within a few hours or a few days are either slow vascular lesions or rapid inflammations. Cases developing in a week or so are inflammations or rapid growths. Cases requiring from six weeks to six months in which to develop are either very slow chronic inflammations, growths of moderate rate of growth, or rapid degenerations. Cases with an onset longer than six months are always either degeneration or a growth of slow development.

The classification of functional neuroses resolves itself largely into a question of whether the case is one of hysteria, neurasthenia, or hypochondria. Whether Janet's subdivision of the neurosis of exhaustion into the more or less acquired neurasthenia proper and the more or less inherited psychasthenia is advantageous complication of our classification or not, is, I think, rather doubtful. But I do believe that we often neglect to draw the valuable distinction between neurasthenia proper and hypochondria. Without attempting to catalogue all the stigmata and accidents of hysteria, all the phobias of neurasthenia, or all the imaginary ills of hypochondria, I shall merely point out that in typical neurasthenia the mind of the patient is mainly obsessed with imaginary dangers from without, while the mind of the classical hypochondriac is obsesses with imaginary dangers from within. Moreover, the neurasthenic is weak from exhaustion, while the hypochondriac is often well-nourished and even robust.

In conclusion, it should be emphasized that perhaps most cases of functional nervous disease are mixed with other functional neuroses, and that it is always possible for the functional ailments to become grafted upon organic maladies in a manner truly calculated to mislead the long-suffering diagnostician.

1027 Candler Building.

